# The Effects of Nutrient Imbalances and Temperature on the Biomass Stoichiometry of Freshwater Bacteria

**DOI:** 10.3389/fmicb.2017.01692

**Published:** 2017-09-08

**Authors:** Katherine N. Phillips, Casey M. Godwin, James B. Cotner

**Affiliations:** ^1^Department of Ecology, Evolution and Behavior, University of Minnesota St. Paul, MN, United States; ^2^Science Department, Saint Paul College Saint Paul, MN, United States; ^3^School of Natural Resources and Environment, University of Michigan Ann Arbor, MI, United States

**Keywords:** temperature, phosphorus, carbon, nitrogen, stoichiometry, nucleic acids, morphology

## Abstract

Two contemporary effects of humans on aquatic ecosystems are increasing temperatures and increasing nutrient concentrations from fertilizers. The response of organisms to these perturbations has important implications for ecosystem processes. We examined the effects of phosphorus (P) supply and temperature on organismal carbon, nitrogen and phosphorus (C, N, and P) content, cell size and allocation into internal P pools in three strains of recently isolated bacteria (*Agrobacterium* sp., *Flavobacterium* sp., and *Arthrobacter* sp.). We manipulated resource C:P in chemostats and also manipulated temperatures from 10 to 30°C. Dilution rates were maintained for all the strains at ~25% of their temperature-specific maximum growth rate to simulate low growth rates in natural systems. Under these conditions, there were large effects of resource stoichiometry and temperature on biomass stoichiometry, element quotas, and cell size. Each strain was smaller when C-limited and larger when P-limited. Temperature had weak effects on morphology, little effect on C quotas, no effect on N quotas and biomass C:N, but had strong effects on P quotas, biomass N:P and C:P, and RNA. RNA content per cell increased with increasing temperature at most C:P supply ratios, but was more strongly affected by resource stoichiometry than temperature. Because we used a uniform relative growth rate across temperatures, these findings mean that there are important nutrient and temperature affects on biomass composition and stoichiometry that are independent of growth rate. Changes in biomass stoichiometry with temperature were greatest at low P availability, suggesting tighter coupling between temperature and biomass stoichiometry in oligotrophic ecosystems than in eutrophic systems. Because the C:P stoichiometry of biomass affects how bacteria assimilate and remineralize C, increased P availability could disrupt a negative feedback between biomass stoichiometry and C availability.

## Introduction

Freshwaters are increasingly recognized as important regulators of exported terrestrial organic carbon through effects on mineralization and burial (Tranvik et al., 2009). Aquatic heterotrophic bacteria play an important role in aquatic systems as the “gatekeepers” to aquatic food webs through effects on the mineralization of nutrients and organic carbon (Cotner and Wetzel, 1992; Cotner and Biddanda, 2002) with important feedbacks to ecosystem productivity and global change processes. Therefore, it is important to understand how heterotrophic bacteria respond to two important and simultaneous anthropogenic drivers of freshwater ecosystem processes: global warming and eutrophication (Baron et al., 2002; Carpenter, 2005).

Temperature plays a critical role in microbial metabolism. Early work by Pomeroy and others indicated that temperature has important effects on interactions between marine heterotrophic bacteria and phytoplankton, with implications for export production and subsequent burial in the polar oceans (Pomeroy and Deibel, 1986). Increasing temperatures decrease the growth efficiency of heterotrophic bacteria in both marine and freshwater ecosystems, leading to higher nutrient regeneration efficiencies at high temperatures (Rivkin and Legendre, 2001; Biddanda and Cotner, 2002; Apple et al., 2006; Hall and Cotner, 2007). Another way that temperature affects nutrient regeneration and carbon cycling by bacteria is through effects on biomass stoichiometry (Cotner et al., 2006; Chrzanowski and Grover, 2008; Hall et al., 2009). Previous work with rapidly growing *Escherichia coli* in a nutrient replete chemostat culture at a constant dilution rate demonstrated that increasing temperature led to decreased P content, and increased carbon (C) to phosphorus (P) ratios, which we argued was partially due to increased translational efficiency of ribosomal RNA at higher temperatures (Dicks and Tempest, 1966; Cotner et al., 2006). This work was consistent with a large survey of ectotherms from microbes to higher plants and animals indicating that P content and RNA content decreased at higher temperatures (Woods et al., 2003).

However, the effects of temperature on bacterial metabolism and stoichiometry can be confusing due to interactive effects of temperature and resource availability and adaptation by communities to a given thermal regime (Hall et al., 2008; Martiny et al., 2016). A problem with interpreting temperature effects on microorganisms is that the maximum growth rate generally increases with temperature, which means that at a fixed dilution rate, increasing temperature would decrease the relative growth rate, i.e., the ratio between realized growth rate and the maximal growth rate. Therefore, experiments that use a fixed dilution rate to examine the effect of temperature run the risk of inadvertently manipulating relative growth rate, which has been shown to affect biomass stoichiometry, element quotas, and growth efficiency (Godwin et al., 2017). Furthermore, the extent to which the realized growth rate, but not the maximal growth rate, increases with temperature is very much dependent on resource availability. To address some of these concerns, we isolated strains of heterotrophic bacteria from lakes in northern Minnesota and examined their macromolecular and biomass elemental content at varying temperatures and nutrient (P) availability. Rather than maintaining a constant dilution rate in chemostats, we determined the maximum growth rate at each temperature and ran the chemostats at 25% of their temperature-specific maximum growth rate, maintaining a uniform relative growth rate by varying the dilution rate at all temperatures. We hypothesized that normalizing the relative growth rate across temperatures would minimize the effects of temperature on stoichiometry but would also allow us to understand how nutrients and carbon affect biomass composition independent of growth rate.

## Materials and methods

Water was collected from three lakes in northern Minnesota (Itasca State Park, Hubbard and Clearwater counties) and bacterial cultures were established using the agar streak plate method onto complex culture media (Difco nutrient agar, cellulose + Difco nutrient agar, or LB agar medium). Individual colonies were picked from plates after visible growth was observed and this process was repeated several times with the goal of isolating individual bacterial strains. Each unique strain was transferred to plates containing defined nutrient rich basic minimal media (BMM; Tanner, 2007) with 23.88 mmol C L^−1^ as glucose and then grown in the same media as broth until late log phase. These cultures were stored at −70°C in 15% glycerol until use. The bacterial strains examined in this study were identified using partial 16S rDNA sequence analysis (Ghosh and LaPara, 2007) as: *Agrobacterium*, sp. (Phylum: Alpha-proteobacteria; Gram negative), *Flavobacterium*, sp. (Phylum: Bacteriodetes; Gram negative), and *Arthrobacter*, sp. (Phylum: Actinobacteria; Gram positive) and were isolated from Deming, Itasca and Elk lakes, respectively. We chose these strains because they represent a wide diversity from classes that are well-represented in freshwaters (Newton et al., 2011), and previous work with two of the strains indicated that they differed in how they regulate their biomass composition (Scott et al., 2012).

Experiments were conducted at 10, 15, 20, 25, and 30°C, which nearly encompasses the annual temperature range experienced in these lakes. Because the maximal growth rate (μ_max_) is temperature dependent (Kuhn et al., 1980), we determined μ_max_ of each strain at each temperature by measuring the optical density at 600 nm during logarithmic growth in batch culture in a nutrient replete medium (in BMM with 2.4 mM phosphate and 19 mM ammonium). We cultured each strain in a complete factorial design of temperature and P availability. At each combination of temperature and P availability, each strain was grown in triplicate chemostat cultures at 25% μ_max_. The phosphate concentration of the medium was manipulated to achieve molar resource C:P ratios (C:P_R_) of 50, 250, and 1,000:1. The sterile 80 mL acid-soaked glass chemostats were mixed continuously with filtered air until the cell densities reached steady state and then measurements were made.

It is known that relative growth rate (proportional to a temperature-specific maximum growth rate) is a strong driver of elemental content and stoichiometry (Godwin et al., 2017). In order to isolate the unknown effect of temperature from the known effect of relative growth rate, we adjusted the dilution rate to a fixed proportion of the maximum growth rate observed at each temperature. If we had used a single dilution rate at all temperatures, the relative growth rate would be different for each temperature. As a result, we would not be able to separate the effect of temperature from relative growth rate.

### Cell abundance and cell morphology

A sample of cells harvested from each chemostat was preserved in formalin (final concentration of 3.7%) and stored at 4°C. The fixed cells were diluted in 118 mM pyrophosphate to reduce clumping. Also, after a 30 min period of shaking at 200 rpm, the cells were sonicated on ice for 2 min in 30-s intervals, then stained with acridine orange, filtered onto 0.2 μm pore-size black polycarbonate filters and examined by epifluorescence microscopy (Hobbie et al., 1977). Cell abundance was estimated by counting a minimum of 10 fields. The bacteria were photographed with a SPOT digital camera (Diagnostics Instruments, Inc) for image analysis. Mean cell length and width were measured for a minimum of 75 randomly selected cells from each chemostat using Image Pro Plus software (Media Cybernetics, version 4.5.1). Cell volume and surface area of *Agrobacterium* sp. and *Flavobacterium* sp. were determined using equations assuming the cell shape was a cylinder capped by two hemispheres (Sun and Liu, 2003). Cell volume and cell surface area of *Arthrobacter* sp. was determined by using equations derived for a prolate spheroid (Sun and Liu, 2003).

### Elemental analysis

Elemental analyses for carbon and phosphorus were performed on biomass samples from the chemostats to assess the elemental composition of each strain. Bacteria were harvested and filtered (<5 ^*^ 10^−5^ atm) onto pre-combusted Whatman GF/F filters for particulate carbon analysis and onto acid-rinsed GF/F filters for particulate phosphorus analysis. All samples were collected and analyzed in triplicate. The particulate organic carbon content was determined using a CHN analyzer (PerkinElmer, model 2400). The P content was determined spectrophotometrically following an acid-persulfate digestion, reacting with molybdenum blue and absorption measurements at 880 nm (APHA, 1992).

### Nucleic acids

Cells from each chemostat were collected on 0.2 μm white polycarbonate filters in triplicate. Bacterial nucleic acids were determined after extraction via sonication and staining with the fluorochrome RiboGreen (Molecular Probes), which reacts with both DNA and RNA (Gorokhova and Kyle, 2002). Bacterial samples on the filters, negative control samples (containing all reagents but no bacteria), and standards of RNA (*E. coli*, Invitrogen) and DNA (calf thymus, Sigma) were prepared for analysis as described in Makino et al. (2003). Two identical 96-well black microplates were prepared with one plate receiving an addition of RNase (Promega; conc in 1X ^*^ TE buffer) before the 30-min digestion period. After the digestion reaction period, 75 μL of RiboGreen was added to both plates followed by a second reaction period of 10 min. The fluorescence of the samples was measured on both plates (excitation 480 nm and emission at 520 nm) on a Micromax 384 plate reader (Fluormax, Horbia Jobin Yvon) using DataMax software. The fluorescence of the digested sample was used to determine DNA concentration and differences between the undigested and digested plates were used to determine RNA concentrations.

### Statistical analysis

The effect of varying C:P_R_ and temperature on the molar ratio of biomass C:P in the replicated chemostats was evaluated using analysis of variance (ANOVA) using the JMP® statistical software (version 11.2). ANOVAs were conducted on biomass parameters with temperature, resource C:P and strain as fixed effects. We did a three-way ANOVA initially and if there were statistically significant interactions, we conducted a two-way ANOVA for each strain (with temperature and P as the independent variables). If there was a significant interaction in the two-way ANOVA, then we did a one-way test for each variable at each level of the other variable. We used a *p*-value of less than or equal to 0.05 to determine statistical significance.

## Results

**Figure 1:**
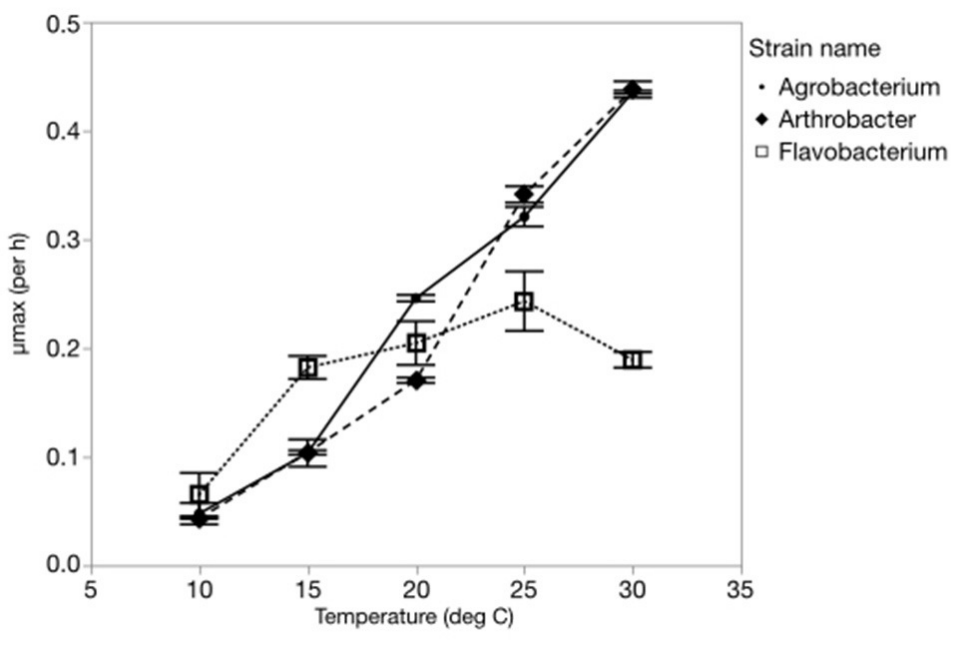
The effects of temperature on the maximum growth rates (μ_max_) of each strain from 10 to 30°C.

The maximum growth rate of the three strains varied greatly with temperature, with all three strains having a μ_max_ <0.08 hr^−1^ at 10°C and a μ_max_ greater or equal to 0.2 hr^−1^ at the highest temperature (Figure 1). Two of the strains (*Agrobacterium* sp. and *Arthrobacter* sp.) had very similar temperature-dependent growth curves with the highest maximum growth rate at the highest temperature used in this study (30°C). However, *Flavobacterium* reached its highest growth rate at 25°C and μ_max_ varied only from 0.07 to 0.25 hr^−1^ from 10 to 30°C.

### Morphology and biovolume

**Figure 2:**
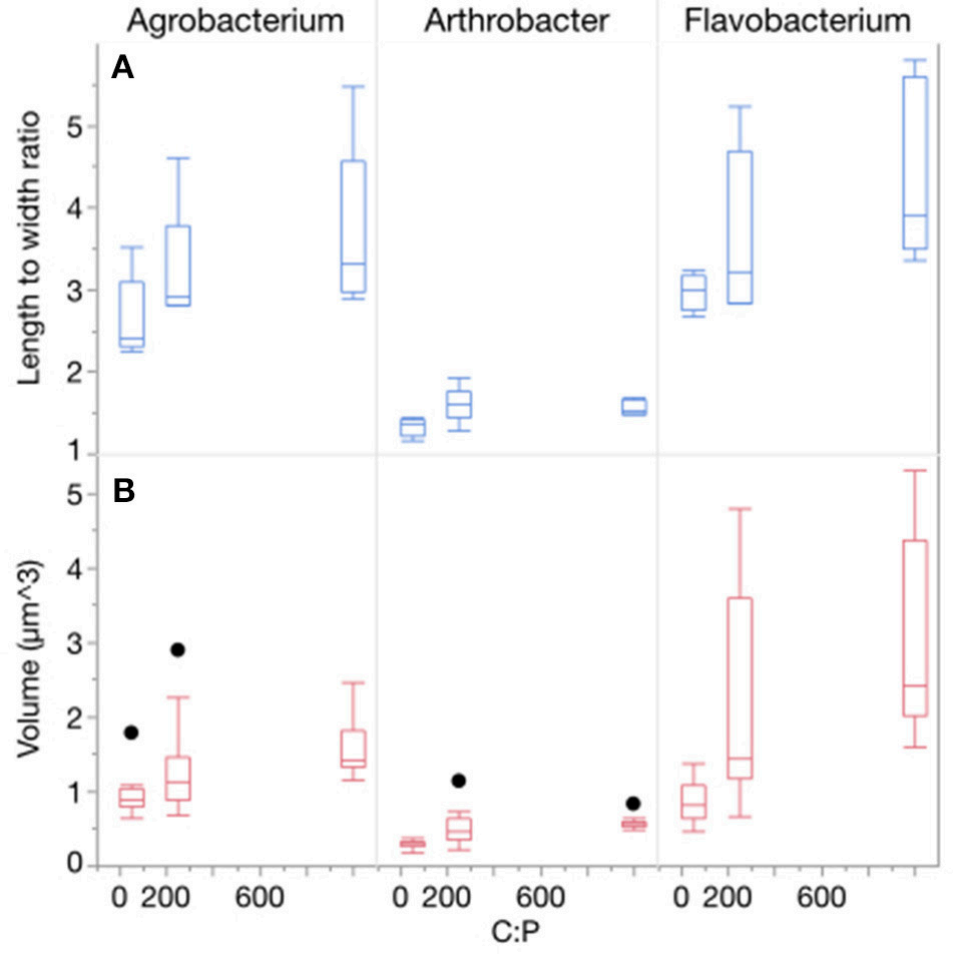
**(A)** Changes in the morphology (length to width ratios) of each strain across C:P supply ratios. Bar depict the median with the 25th and 75th percentiles and error bars are the 95% confidence interval with outliers represented by points. **(B)** Changes in the biovolume (μm^3^) of each strain across C:P supply ratios.

Three-way ANOVAs on cell volume and length-to-width ratios indicated that there were significant effects of both C:P_R_ ratio and different strains but not temperature (Table 1, Figure 2, Table S1) on morphology. There was a strain^*^temperature interaction with length to width ratios but not biovolume, and there was a strain^*^C:P_R_ interaction with both length to width ratios and biovolume. Two-way ANOVAs revealed significant effects of temperature only on the biovolume of *Arthrobacter* and the length to width ratio of both *Agrobacterium* and *Arthrobacter* (Table 1, Table S1). C:P_R_ ratio had significant effects on both morphometric variables in all three strains and there were no interactions between C:P_R_ ratio and temperature (Table 1, Table S1). As C:P_R_ increased, the biovolume and length to width ratios also increased. There was a significant effect of C:P_R_ on both biovolume and L:W ratios in the 3-way (*p* < 0.0001) and two-way ANOVAs (all *p* < 0.006). There was a great deal of variability in both the volume and the L:W ratios among the different strains, however (Figure 2). There were large differences in the mean length: width ratios of the three strains with *Flavobacterium* sp. and *Agrobacterium* sp., having higher ratios (3.68 and 3.20, respectively) than *Arthrobacter* sp. (1.50). *Arthrobacter* also had the lowest volume (mean = 0.46 μm^3^) followed by *Agrobacterium* (1.30 μm^3^) and *Flavobacterium* (2.03 μm^3^).

**Table 1:** Three-way and two-way ANOVAs.

	**Three-way ANOVA**						***Agrobacterium***			***Arthrobacter***			***Flavobacterium***		
	**S**	**T**	**P**	S^*^T	S^*^P	T^*^P	**T**	**P**	T^*^P	**T**	**P**	T^*^P	**T**	**P**	T^*^P
C quota	^***^	ns	^***^	ns	^**^	^***^	ns	^***^	^***^	ns	^***^	ns	^*^	^***^	^**^
N quota	^***^	ns	^***^	ns	^*^	ns	ns	^***^	^*^	ns	^***^	ns	ns	^***^	^*^
P quota	ns	^***^	^**^	ns	ns	^**^	ns	^**^	ns	^***^	ns	ns	^*^	ns	^*^
Volume	^***^	ns	^***^	^*^	^**^	ns	ns	^*^	ns	^*^	^***^	ns	ns	^***^	ns
L:W	^***^	ns	^***^	^*^	^**^	ns	^**^	^***^	ns	^***^	^**^	ns	ns	^***^	ns
C:N	^***^	ns	^***^	ns	ns	ns	ns	^***^	ns	ns	ns	ns	ns	^**^	ns
N:P	^***^	^***^	^***^	ns	^*^	^***^	^**^	^***^	ns	ns	^***^	ns	^*^	^***^	^*^
C:P	^**^	^***^	^***^	ns	^*^	^***^	^**^	^***^	^*^	^**^	^***^	ns	^**^	^***^	^*^
RNA	^***^	^*^	^***^	ns	ns	ns	^*^	^*^	ns	ns	^*^	ns	ns	^*^	ns
DNA	^***^	ns	ns	ns	ns	ns	ns	^**^	ns	ns	ns	ns	ns	ns	ns
Total ns	1	6	1	8	4	6	6	0	7	6	3	10	6	2	5
Total sig	9	4	9	2	6	4	4	10	3	4	7	0	4	8	5

Statistically insignificant results are denoted by “ns” and significant results (p < 0.05) are denoted with asterisks, with more asterisks representing lower p-values. Units for each parameter are the same as discussed in the paper. S, strain; T, temperature; P, C:P_R_; S^*^T, strain by temperature interaction; S^*^P, strain by C:P_R_ interaction; T^*^P, temperature by C:P_R_ interaction. There were no three-way interactions (S^*^T^*^P). We summed the non-significant and significant observations at the bottom of the table.

### Internal pools and ratios

**Figure 3:**
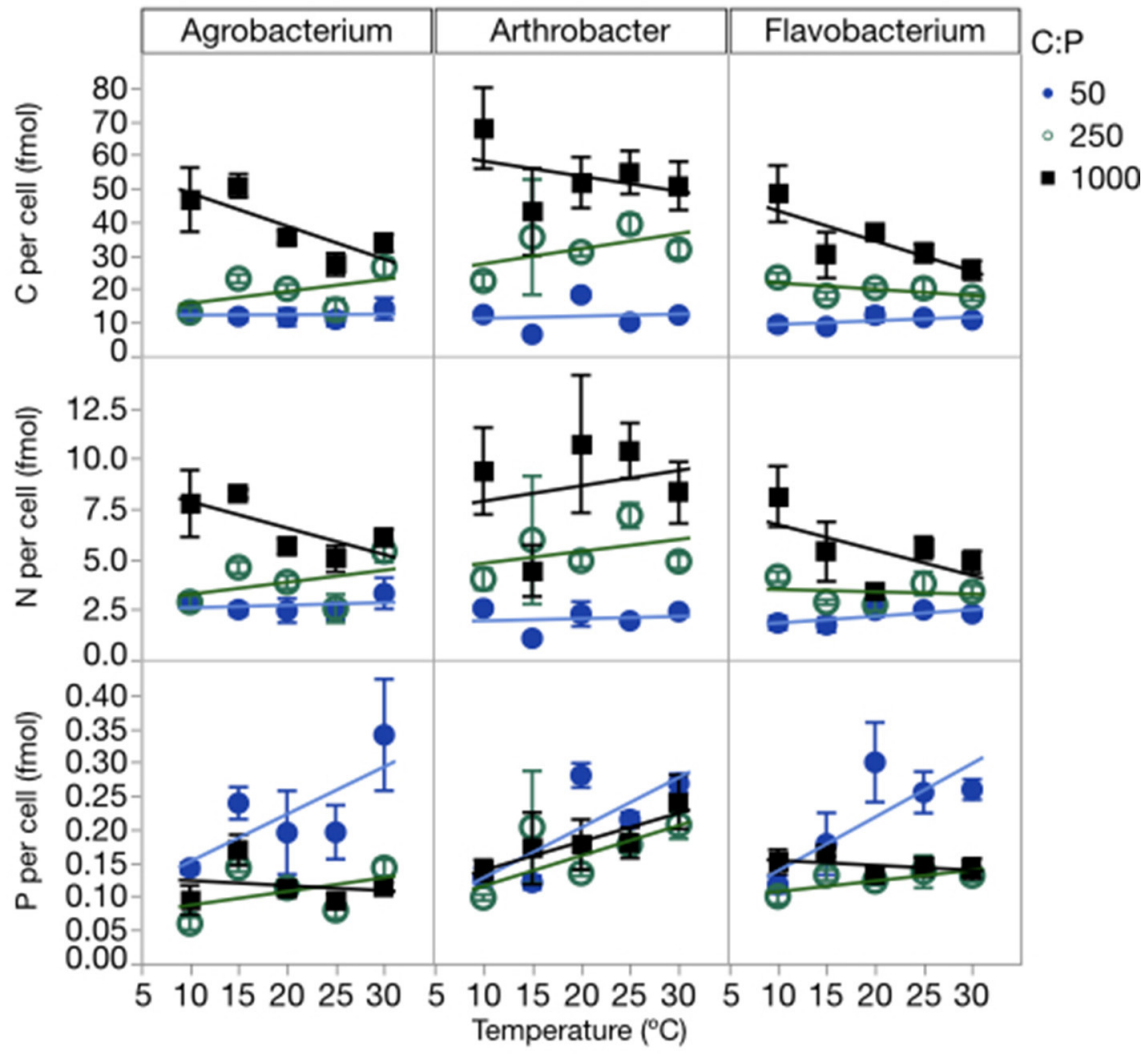
Changes in cellular C, N, and P quotas of each strain across temperatures, with different symbols for each level of C:P_R_. Error bars represent one standard error of the mean.

Temperature and P availability interactively affected biomass element content. Consistent with differences in the sizes of the three strains, the strains also differed in terms of C and N content per cell, but not P content per cell (Table 1, Figure 3, Table S2; C quota: *p* < 0.0001; N quota: *p* = 0.0003; P quota: *p* = 0.068). Temperature had no effect on C and N cell quotas but it did affect P quotas (*p* < 0.0001), with increasing quotas with temperature increases and at the lowest C:P_R_ (Table 1, Figure 3). C, N, and P cell quotas were strongly affected by C:P_R_ and there were significant interactions between temperature and C:P_R_ for C, N, and P quotas, especially C and N quotas for *Agrobacterium* and *Flavobacterium* (Table 1). C:P_R_ had effects on the C and N quotas of all three strains. P quotas were affected by different factors among the strains. C:P_R_ had a significant effect on P quotas for *Agrobacterium* (*p* = 0.006) while the other two strains were affected more by temperature (*p* < 0.0001 and *p* = 0.0069 in *Arthrobacter* and *Flavobacterium*, respectively). There was also a temperature^*^C:P_R_ interaction in *Flavobacterium*.

**Figure 4:**
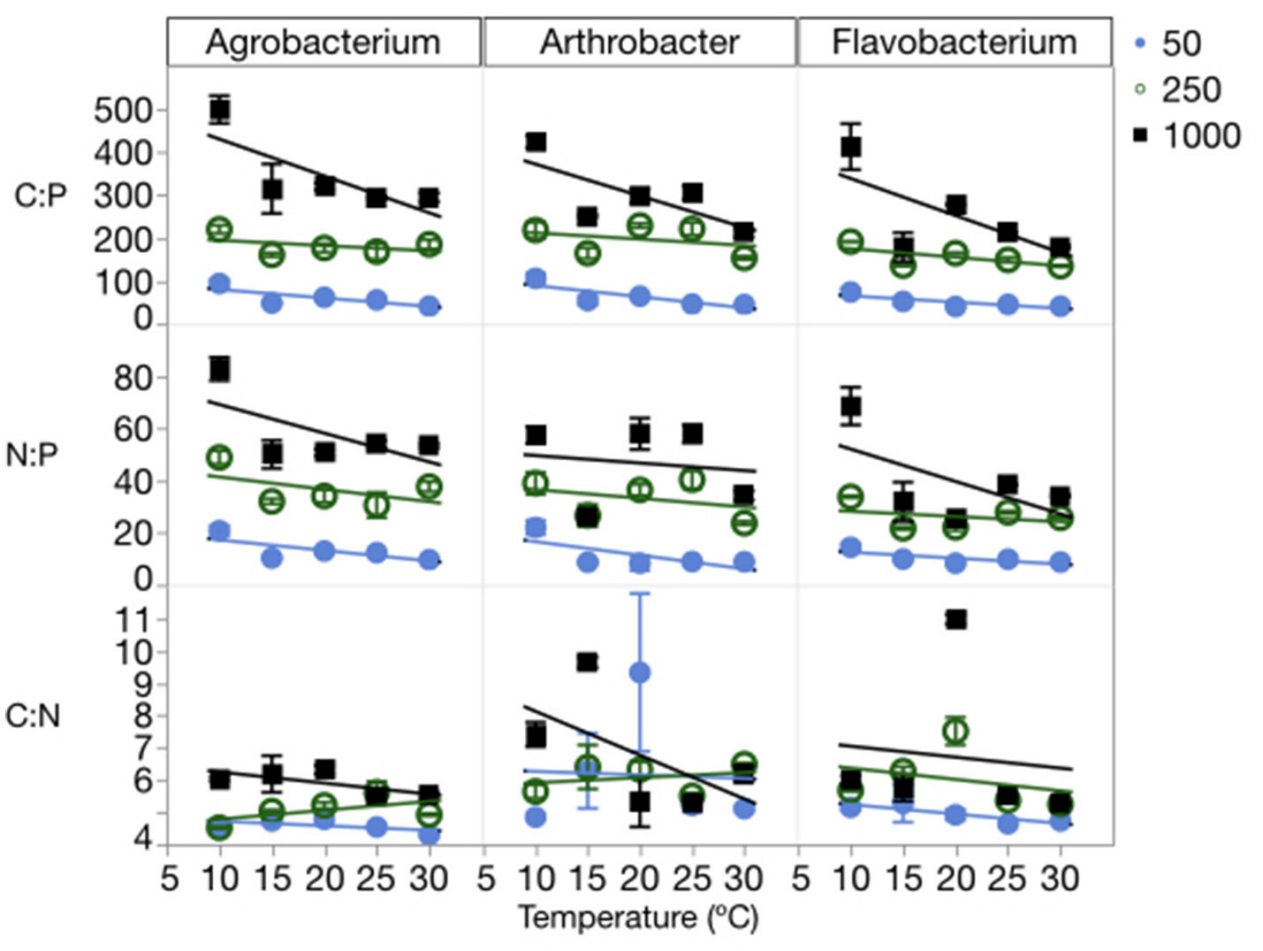
Changes in the biomass stoichiometry (molar ratios of C:P, C:N, and N:P) of each strain across temperatures, with different symbols for each level of supply C:P. Error bars represent one standard error of the mean. The three-way ANOVA for C:N showed significant effects of strain and supply C:P only (*p* < 0.001). The ANOVA for C:P showed significant effects of strain, temperature, supply C:P, an interaction between strain and supply C:P, and an interaction between temperature and supply C:P (*p* < 0.01). The ANOVA for N:P showed significant effects of strain, temperature, supply C:P, and an interaction between strain and supply C:P (*p* < 0.05).

The changes in cell quotas were not consistent among C, N, and P, leading to substantial variation in the effects of temperature and P on biomass stoichiometry (Table 1, Table S3). The biomass C:P (C:P_B_) ratios were high at a C:P_R_ of 1000:1, with all three strains having ratios near 500:1 at the lowest temperatures and decreasing with increased temperature (Figure 4). The median biomass C:P for the three strains was similar, varying from 144 in *Flavobacterium* to 181 in *Agrobacterium* and 202 *Arthrobacter*. Although, biomass C:P decreased with temperature at all levels of C:P_R_, the response of biomass C:P to temperature was strongest at the highest C:P_R_, indicating that biomass C:P_B_ ratios were the most sensitive to temperature changes when P was least available. A similar pattern was observed for N:P but less so for C:N. The mean N:P biomass ratio for the three strains varied from 25 in *Flavobacterium* to 31 in *Arthrobacter* and 36 in *Agrobacterium*. The mean C:N_B_ for the three strains varied much less among strains, with a mean of 5.9 in *Flavobacterium, to* 6.3 in *Arthrobacter* and 5.1 in *Agrobacterium*.

Consistent with the cell quota observations, temperature did not affect biomass C:N ratios, rather only biomass N:P and C:P ratios (Figure 4, Table 1). There were significant effects of temperature on both C:P_B_ and N:P_B_ in all three strains, with the exception of *Arthrobacter* biomass N:P. C:P_R_ also had significant effects on all three biomass ratios in each of the strains, with the exception of the C:N of *Arthrobacter*.

**Figure 5:**
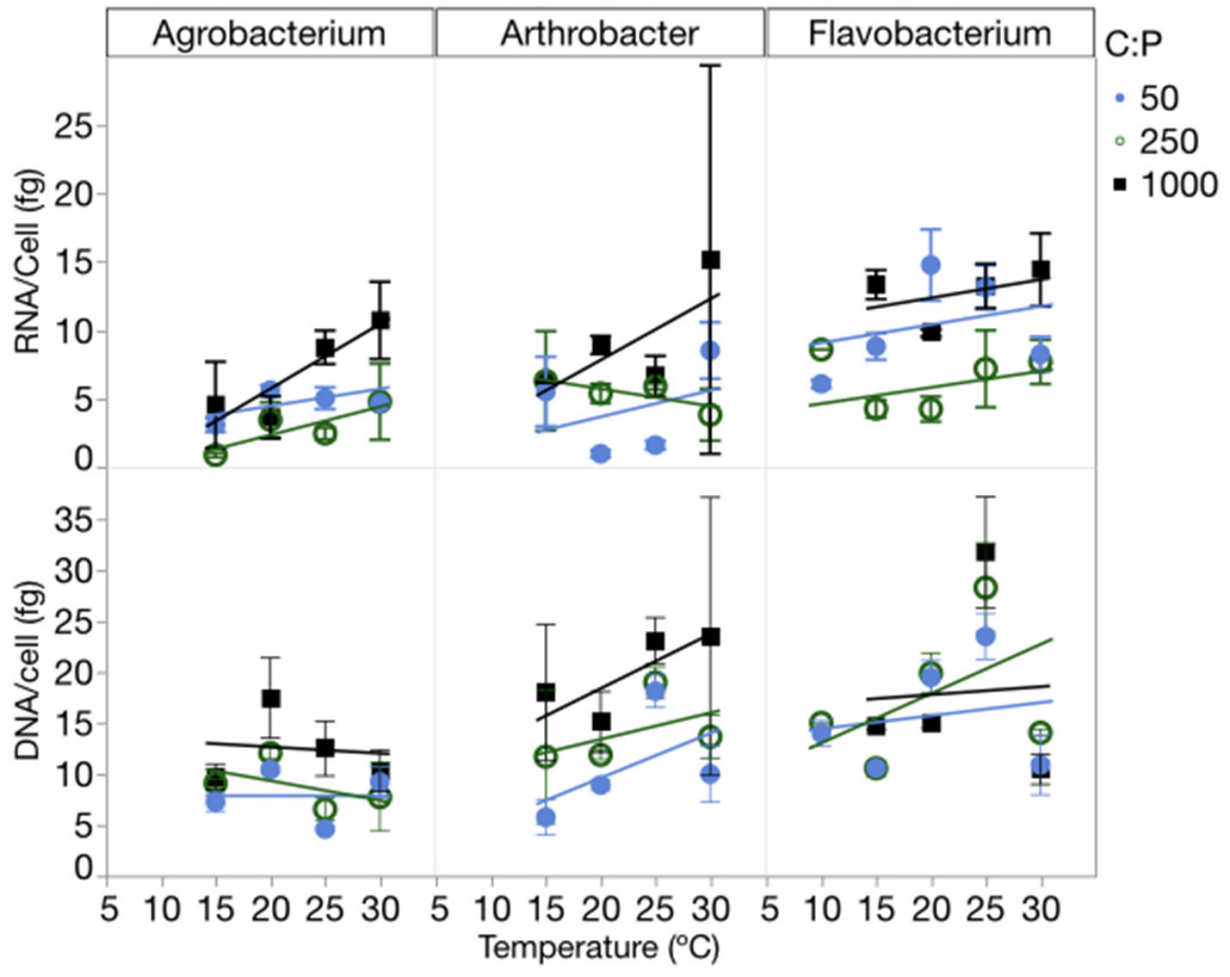
Changes in the RNA and DNA content (fg cell^−1^) of each strain across temperatures, with different symbols for each level of supply C:P. Error bars represent one standard error of the mean.

RNA per cell varied by strain, temperature and C:P_R_, while DNA per cell varied only among strains (Figure 5, Table 1, Table S4). There were no interactions for either of these factors. RNA per cell was highest when C:P_R_ was highest (lowest P supply) in two of the strains (*Agrobacterium* and *Flavobacterium)*, perhaps due to increased cell size when P was least available. In addition, RNA per cell increased with increasing temperatures in *Agrobacterium* and *Arthrobacter*. *Agrobacterium, Arthrobacter*, and *Flavobacterium* had mean values of 4.8, 4.7, and 9.5 fg RNA cell^−1^, respectively. Across all levels of temperature and C:P_R_, *Agrobacterium, Arthrobacter*, and *Flavobacterium* had mean values of 9.9, 9.8, and 16.8 fg DNA cell^−1^, respectively, giving RNA/DNA ratios of 0.48, 0.48, and 0.56, respectively.

**Figure 6:**
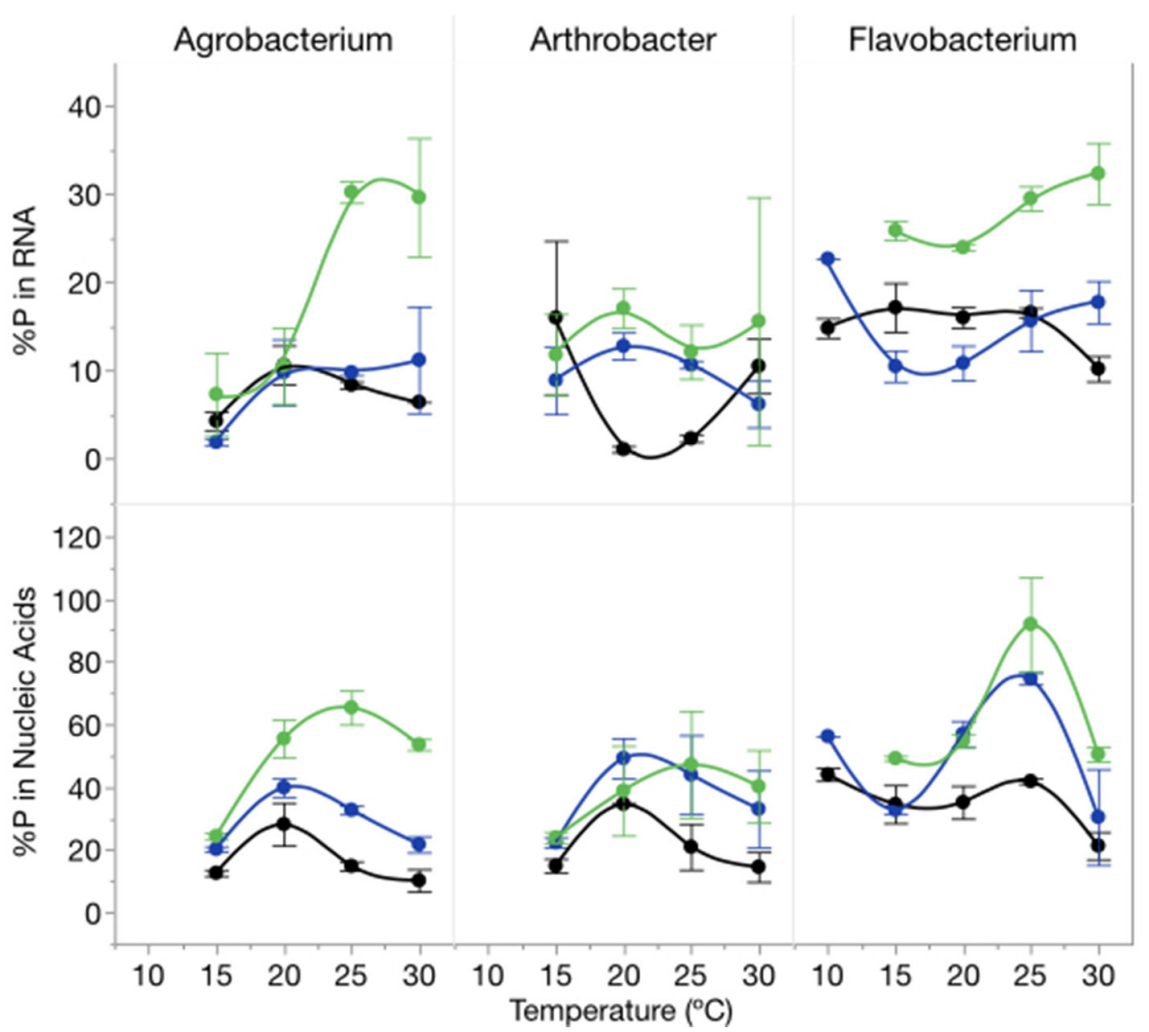
Changes in %P in RNA (top panel) and %P in nucleic acids (bottom panel) for each strain across temperatures and C:P_R_ with different symbols for each level of supply C:P (black-50:1; blue: 250:1; green: 1000:1). Error bars represent one standard error of the mean.

The proportion of cellular P in nucleic acids (RNA and DNA) increased with increasing C:P_R_ particularly at 1,000:1 and varied among strains (Figure 6). There was an effect of temperature on the proportion of total P in RNA (*p* = 0.0139) but no effect on total nucleic acid content per cell. The percentage of P in nucleic acids varied from a low of 10% (*Agrobacterium*, high P supply and 10°C) to 92% (*Flavobacterium*, low P supply, 25°C) with mean percentages of 32% for both *Agrobacterium* and *Arthrobacter* and 48% for *Flavobacterium*.

## Discussion

Here we show that temperature and resource stoichiometry both affect the biomass stoichiometry and physiology of aquatic heterotrophic bacteria and illustrate that these factors had a strong interactive effect that was independent of the relative growth rate. We observed important effects of C:P supply on all of these parameters, particularly cell size and shape, as well as internal pools of C, N, and RNA. One of the key observations was that the biomass C:P stoichiometry in these three strains was quite responsive to varying temperatures and nutrient supply rates, going from about 40:1 at high temperatures and high P availability to over 500:1 at cooler temperatures and low P, confirming recent observations that both bacterial strains and bacterial communities can be quite flexible in their C and P content (Hochstädter, 2000; Cotner et al., 2010; Scott et al., 2012; Godwin and Cotner, 2014, 2015).

Perhaps as important as the ability of these strains to vary internal P content was their capacity to vary C content, primarily mediated via changes in cell size (Garcia et al., 2016). Temperature has important influences on the stoichiometry of ectotherms as well as endotherms in freshwater, terrestrial and marine systems (Gillooly et al., 2002; Woods et al., 2003; Reich and Oleksyn, 2004; Borer et al., 2013). The general pattern of decreased cell size at higher temperature (Bergmann's Rule) likely mediates many of these interactions but our study seems to indicate that, at least in heterotrophic bacteria, decreased cell size at increased temperatures may be mediated in part by the effects of temperature on relative growth rates. Specifically, we only observed effects of temperature on the morphology of *Arthrobacter*.

Related to the morphology of these three strains, it is curious that we more frequently found temperature and C:P_R_ effects on *Agrobacterium* and *Flavobacterium* (both Gram negative) than on *Arthrobacter* which is Gram positive. It is speculative given the small number of strains examined here, but the thicker and more rigid structure of Gram positive bacterial cell walls may constrain their morphology (Cabeen and Jacobs-Wagner, 2005), thereby restricting their capacity to significantly change cell quotas and stoichiometry. To the best of our knowledge, these connections between cell wall structure and stoichiometry in bacteria have not been examined but could provide an alternative explanation for why some strains are more homeostatic than others (Godwin and Cotner, 2015).

This work provides important insights on the growth rate hypothesis (GRH), a tenet of ecological stoichiometry (Elser et al., 2000). By maintaining a uniform relative growth rate while changing the temperature, we were able to observe how temperature and nutrient limitation affect biomass composition independent of relative growth rate. Cell size and morphology were strongly affected by nutrient limitation, i.e., C:P_R_ ratios, with accompanying changes in cell quotas and stoichiometry in all three strains. Moving forward, it is important to keep in mind that limitation by different nutrients can impose changes in morphology, with effects on stoichiometry, that are independent of growth rate. In contrast, temperature had mixed effects on morphology (effects on cell volume and length to width ratios in *Arthrobacter*, effect on cell volume for *Agrobacterium*, but no effects on *Flavobacterium*), but had effects on P quotas in two of the three strains and effects on biomass N:P in two strains and C:P ratios in three strains. The fact that these effects were observed at uniform relative growth rate indicates that there can be important effects of nutrients and temperature that are unrelated to the GRH.

By minimizing the influence of relative growth rate in the present study, we observed what more closely represents the effects of temperature on internal pools of C, N, and P (Table 1, Table S2). Relative growth rate is an important concept particularly for ecological stoichiometry because it describes what portion of the organismal biomass should be focused on growth vs. other cellular activities (resource acquisition; structural materials, maintenance, etc.; Klausmeier et al., 2004). As the relative growth rate increases, the concentration of P-rich ribosomes should increase and therefore the C:P and N:P ratios should decrease (Elser et al., 2003). The GRH (Elser et al., 2000) predicts that RNA content is proportional to growth rate, so it was somewhat surprising that we observed increased RNA with increasing temperatures at uniform relative growth rates which may have been due to changes in cell size or differences in ribosome translation efficiency or degradation that vary with temperature (Dicks and Tempest, 1966) and/or nutrient limitation (Deutscher, 2003).

### Temperature and relative growth rate

As mentioned above, there were mixed effects of temperature on elemental quotas and stoichiometry. In previous studies, temperature has been shown to have important effects on cell size and stoichiometry in diverse organisms, from bacteria to fish and terrestrial, freshwater and marine systems (Daufresne et al., 2009; Forster et al., 2012; Borer et al., 2013; Morán et al., 2015). Specifically, past studies have shown that increasing temperature generally leads to *increased* C:P, N:P, and C:N ratios (Woods et al., 2003; Cotner et al., 2006; Martiny et al., 2013), but in the present study we found that increasing temperatures often led to *decreased* biomass C:P and N:P ratios (*p* < 0.05 for N:P in two strains and *p* < 0.05 for C:P in all strains). This contradiction is likely due to differences in growth and temperature manipulations. In a chemostat at a constant dilution rate, as temperature increases, the relative growth rate will decrease due to a uniform realized growth with increasing μ_max_, in contrast to the uniform relative growth in the present experiments.

Of the previous studies that have examined the effects of temperature on biomass stoichiometry, Chrzanowski and Grover (2008) manipulated both the dilution rate and temperature in a chemostat study using *Pseudomonas*. They observed that growth rate had a larger effect on biomass stoichiometry than did temperature and this observation was one of the motivations for why we maintained uniform relative growth rates in the present study. However, unlike the present study, Chrzanowski and Grover (2008) observed that cell quotas for C, N, and P were highest at either low temperature and low dilution rate or high temperature and high dilution rate. At low dilution rates, C, N, and P content decreased with increasing temperatures, but in the present study there was no significant effect of temperature on C and N cell quotas, while P cell quotas increased with increasing temperatures, particularly when P availability was high. Also, in their study, at high dilution rates, C, N, and P quotas increased with temperature, similar to our observations with *E. coli* for C and P (Makino et al., 2003).

The lack of a consistent effect of temperature on cell size (Table S1) and quotas (Table S2) was also surprising in light of past work showing an inverse relationship between temperature and cell size. Among species, there is a general decline in maximal growth rates and metabolic rates with increasing size (Sommer, 1989; Niklas and Enquist, 2001) but variation within a species is influenced by the relative growth rate, due to the fact that growth and structural components scale differently with size (Clark et al., 2013). As relative growth rates increase, single cell organisms tend to become larger and more dilute internally (Kempes et al., 2012), whereas increasing temperatures tend to make cells smaller (Woods et al., 2003; Morán et al., 2015). We have recently observed that there is a great deal of variability in how different bacterial strains regulate their C:P and N:P ratios (Godwin and Cotner, 2015) and some of the capacity to do this may be coupled with the capacity of cells to change their morphology.

An obvious question that needs to be addressed is whether, in natural systems the effects of temperature on organism stoichiometry are mediated largely through the effects of temperature on cell size and thus, internal pools as we observed here, or through effects on relative growth rate, as observed in our recent work (Godwin et al., 2017). Biomass stoichiometric ratios (C:P, N:P, or C:N) generally decrease with latitude, a pattern that has been documented in marine plankton (Martiny et al., 2013), multiple ecosystem types (Borer et al., 2013), plant leaf tissue (Reich and Oleksyn, 2004; Kerkhoff et al., 2005), marcrophyte biomass (Xia et al., 2014), and freshwater plankton (Dobberfuhl and Elser, 2000), but many variables other than temperature change along a latitudinal gradient (Martiny et al., 2016), complicating interpretation of these data.

### C:P supply

Although temperature had important effects on several internal pools and some stoichiometric ratios, C:P_R_ affected nearly all of these parameters. This result suggests that, across the range of temperatures and C:P_R_, nutrient availability was the more important determinant of bacterial stoichiometry and physiology at uniform relative growth rates. While it is not surprising that C:P_R_ had a strong effect on the C:P and N:P ratios of the bacterial strains (and to a less extent C:N), the temperature^*^C:P_R_ interaction suggests that temperature modified the response to C:P_R_ (Figure 3). Specifically, biomass C:P was more responsive to differences in C:P_R_ at the lowest temperatures.

Some of the effect of supply stoichiometry on biomass stoichiometry and elemental quotas was attributable to changes in RNA content. There was an increase in RNA content at the highest C:P supply ratio (Figure 5) which is similar to other studies looking at the GRH when P was not limiting (Elser et al., 2003). This suggests that there may be significant changes in RNA unrelated to the growth rate. One possibility is that changes in RNA were caused by changes in cell size. If the cellular ribosome concentration varied little with C:P supply ratios, but there were increases in cell size, it would have increased the RNA content per cell. Biovolumes increased with increasing C:P supply ratios from 56% for *Agrobacterium*, and 108% for *Arthrobacter*, to 255% for *Flavobacterium*.

Temperature had more significant effects on the P quotas of the three strains (*p* < 0.05 for *Arthrobacter* and *Flavobacterium* and *p* = 0.08 in *Agrobacterium*) than did C:P_R_, while C:P_R_ had stronger effects on C and N cell quotas (Figure 3, Table 1). These differences were likely due in a large part to the strong effects that C:P_R_ had on morphology in all three strains (Table 1). The lack of a strong C:P_R_ effect on P quotas likely reflects the compensating effects of P availability on cell volume. Higher P availability leads to decreased cell volume but higher internal concentrations of some pools, while decreased P availability has the opposite effect, leading to a “buffered” P quota per cell. Similar observations have been made by others examining the effects of C and P limitation on cell morphology with P limitation leading to larger and more elongate cells (Løvdal et al., 2008; Godwin and Cotner, 2015). But Løvdal et al. also noted in their study of *Vibrio splendidus* that C, N, and P scaled differently, with larger cells having proportionately less P than C and N.

### Implications for climate change and eutrophication

Temperature is a fundamental environmental variable that affects the behavior of everything from molecules to ecosystems. The metabolic theory of ecology (Brown et al., 2004) demonstrated that metabolic rates vary in predictable ways based on organismal size (mass) and temperature. In a literature survey of organisms from bacteria and algae to plants and animals, Woods et al. (2003) observed that most organisms were more N-, P-, and RNA-rich at colder temperatures. More recently, it was shown that bacterial size (Morán et al., 2010) and plankton stoichiometry (Martiny et al., 2013) in the oceans may both be influenced by temperature. Although temperature has effects on both metabolism and biomass composition, the present study shows that there are important effects of temperature on stoichiometry independent of growth rates. This mechanism is not widely recognized in biogeochemical modeling, but has important implications for predicting how the pools and fluxes will respond to warming temperatures and increased availability of nutrients.

One thing that was clear in examining the effects of temperature on biomass stoichiometry was that there was much greater change in stoichiometry at different temperatures when P was most limiting. In particular, C:P and N:P slopes relative to temperature were much steeper at the lowest P availability. These results imply that temperature effects on nutrient cycling are likely to be greatest in the least productive (oligotrophic) ecosystems such as large lakes and the open ocean. Furthermore, increased eutrophication in freshwater and marine ecosystems is likely to dampen feedbacks between climate change and biogeochemical processing, due to this decreased sensitivity at high nutrient levels.

## Author contributions

JC designed the research plan and wrote the manuscript; CG implemented portions of the research and wrote the manuscript and KP helped design the research plan, implement the research plan and helped writing the manuscript.

### Conflict of interest statement

The authors declare that the research was conducted in the absence of any commercial or financial relationships that could be construed as a potential conflict of interest. The reviewer RM and handling Editor declared their shared affiliation.
