# Supplementary material for: The Effects of Nutrient Imbalances and Temperature on the Biomass Stoichiometry of Freshwater Bacteria
Source: Front Microbiol. 2017 Sep 8;8:1692. doi: 10.3389/fmicb.2017.01692 (PMC5596061; doi:10.3389/fmicb.2017.01692)
Supplement: Supplementary file 1 [file Table1.PDF]

Supplemental Table 1. ANOVA results for cell volume and length: width ratios.

| | Cell Volume (df, F, p-value) | Cell L:W (df, F, p-value) |
|---|---|---|
| Three-Way ANOVA | $df_{error}$=141 | $df_{error}$=141 |
| Strain | 2, 57.8, <***0.0001*** | 2, 154, ***<0.0001*** |
| Temperature | 1, 0.15, 0.6996 | 1, 2.28, 0.1330 |
| Supply C:P | 1, 43.2, ***<0.0001*** | 1, 40.9, ***<0.0001*** |
| Strain*Temperature | 2, 0.66, 0.5183 | 2, 4.61, ***0.0115*** |
| Strain*C:P | 2, 14.5, ***<0.0001*** | 2, 7.59, ***0.0007*** |
| Temperature *C:P | 1, 0.80, 0.3718 | 1, 0.03, 0.8731 |
| Strain*Temperature*C:P | 2, 0.14, 0.8711 | 1, 2.60, 0.0780 |

| Two-Way ANOVAs | | Agrobacterium (df, F, p-value) | Arthrobacter (df, F, p-value) | Flavobacterium (df, F, p-value) |
|---|---|---|---|---|
| | | $df_{error}$=41 | $df_{error}$=41 | $df_{error}$=41 |
| Cell Volume | Temperature | 1, 0.99, 0.3248 | 1, 4.45, ***0.0399*** | 1, 0.37, 0.5474 |
| | Supply C:P | 1, 8.39, ***0.0060*** | 1, 26.5, ***<0.0001*** | 1, 29.8, ***<0.0001*** |
| | Temperature *C:P | 1, 10, 0.7537 | 1, 2.54, 0.1176 | 1, 0.40, 0.5274 |
| Cell L:W | Temperature | 1, 8.95, ***0.0047*** | 1, 14.8, ***0.0003*** | 1, 0.03, 0.8751 |
| | Supply C:P | 1, 13.8, ***0.0006*** | 1, 10.61, ***0.0020*** | 1, 20.0, ***<0.0001*** |
| | Temperature *C:P | 1, 1.89, 0.1769 | 1, 2.45, 0.1240 | 1, 1.61, 0.2101 |
