# Supplementary material for: The Effects of Nutrient Imbalances and Temperature on the Biomass Stoichiometry of Freshwater Bacteria
Source: Front Microbiol. 2017 Sep 8;8:1692. doi: 10.3389/fmicb.2017.01692 (PMC5596061; doi:10.3389/fmicb.2017.01692)
Supplement: Supplementary file 2 [file Table2.PDF]

Table 2. ANOVA results for cell quotas.

	C Quota (df, F, p-value)	N Quota (df, F, p-value)	P Quota (df, F, p-value)
Three-Way ANOVA	df_{error}=123	df_{error}=123	df_{error}=123
Strain	2, 17.6, ***<0.0001***	2, 8.7, ***0.0003***	2, 2.75, 0.0676
Temperature	1, 2.2, 0.1373	1, 0.01, 0.9367	1, 28.6, ***<0.0001***
Supply C:P	1, 235, ***<0.0001***	1, 120, ***<0.0001***	1, 9.00, ***0.0032***
Strain*Temperature	2, 0.80, 0.4512	2, 1.19, 0.3062	2, 1.57, 0.2125
Strain*C:P	2, 6.32, ***0.0024***	2, 4.87, ***0.0092***	2, 2.14, 0.1209
Temperature *C:P	1, 13.1, ***0.0004***	1, 2.78, 0.0981	1, 11.2, ***0.0011***
Strain*Temperature*C:P	2, 0.31, 0.7357	1, 1.76, 0.1796	2, 0.93, 0.3972

Two-Way ANOVAs		Agrobacterium (df, F, p-value)	Arthrobacter (df, F, p-value)	Flavobacterium (df, F, p-value)
		df_{error}=41	df_{error}=41	df_{error}=41
C Quota	Temperature	1, 2.04, 0.1610	1, 0.01, 0.9404	1, 6.57, ***0.0142***
	Supply C:P	1, 120, ***<0.0001***	1, 67, ***<0.0001***	1, 113, ***<0.0001***
	Temperature *C:P	1, 12.7, ***0.0009***	1, 1.10, 0.2996	1, 11.1, ***0.0019***
N Quota	Temperature	1, 0.55, 0.4613	1, 0.67, 0.4178	1, 1.66, 0.2045
	Supply C:P	1, 73, ***<0.0001***	1, 36.5, ***<0.0001***	1, 49, ***<0.0001***
	Temperature *C:P	1, 7.8, ***0.0079***	1, 0.15, 0.7011	1, 6.16, ***0.0173***
P Quota	Temperature	1, 3.21, 0.0807	1, 27.0, ***<0.0001***	1, 7.93, ***0.0069***
	Supply C:P	1, 8.40, ***0.0060***	1, 0.05, 0.8285	1, 3.08, 0.0854
	Temperature *C:P	1, 3.38, 0.0734	1, 0.99, 0.3245	1, 8.57, ***0.0051***

One-Way ANOVAs		Agrobacterium (df, p-values)	Flavobacterium (df, p-value)
		df_{error}=41	df_{error}=41
C Quota	Temperature	1, **p<0.05 only at $C:P_R$=1000**	1, **p<0.05 only at $C:P_R$=1000**
	Supply C:P	1, **p<0.05 at all temperatures**	1, **p<0.05 at all temperatures**
N Quota	Temperature	1, **p<0.05 only at $C:P_R$=1000**	1, p>0.05 at all $C:P_R$s
	Supply C:P	1, **p<0.05 at all temperatures**	1, **p<0.05 at all temperatures**
P Quota	Temperature	NA	1, **p<0.05 only at $C:P_R$=50**
	Supply C:P	NA	1, **p<0.05 only at 10°C**
