# Supplementary material for: The Effects of Nutrient Imbalances and Temperature on the Biomass Stoichiometry of Freshwater Bacteria
Source: Front Microbiol. 2017 Sep 8;8:1692. doi: 10.3389/fmicb.2017.01692 (PMC5596061; doi:10.3389/fmicb.2017.01692)
Supplement: Supplementary file 3 [file Table3.PDF]

Supplement Table 3. ANOVA results for biomass stoichiometry.

	Biomass C:N (df, F, p-value)	Biomass N:P (df, F, p-value)	Biomass C:P (df, F, p-value)
Three-Way ANOVA	df_{error}=123	df_{error}=123	df_{error}=123
Strain	2, 8.5, ***0.0003***	2, 10.6, ***<0.0001***	2, 7.7, ***0.0007***
Temperature	1, 2.9, 0.0892	1, 16.0, ***0.0001***	1, 35.1, ***<0.0001***
Supply C:P	1, 18.7, ***<0.0001***	1, 216, ***<0.0001***	1, 393, ***<0.0001***
Strain*Temperature	2, 0.45, 0.6380	2, 0.37, 0.6922	2, 0.01, 0.9905
Strain*C:P	2, 0.85, 0.4322	2, 3.62, ***0.0298***	2, 5.0, ***0.0080***
Temperature *C:P	1, 2.6, 0.1094	1, 3.10, 0.0810	1, 19.5, ***<0.0001***
Strain*Temperature*C:P	2, 1.3, 0.2872	1, 1.52, 0.2231	2, 0.17, 0.8435

Two-Way ANOVAs		Agrobacterium (df, F, p-value)	Arthrobacter (df, F, p-value)	Flavobacterium (df, F, p-value)
		df_{error}=41	df_{error}=41	df_{error}=41
Biomass C:N	Temperature	1, 0.63, 0.4337	1, 1.41, 0.2415	1, 1.14, 0.2913
	Supply C:P	1, 76.6, ***<0.0001***	1, 1.30, 0.2604	1, 8.99, ***0.0046***
	Temperature *C:P	1, 3.2, 0.0801	1, 2.83, 0.1003	1, 0.00, 0.9682
Biomass N:P	Temperature	1, 9.2, ***0.0041***	1, 2.2, 0.1499	1, 6.9, ***0.0118***
	Supply C:P	1, 127, ***<0.0001***	1, 50.3, ***<0.0001***	1, 56.2, ***<0.0001***
	Temperature *C:P	1, 2.1, 0.1592	1, 0.10, 0.7593	1, 5.00, ***0.0310***
Biomass C:P	Temperature	1, 12.8, ***0.0009***	1, 10.4, ***0.0025***	1, 12.1, ***0.0012***
	Supply C:P	1, 211, ***<0.0001***	1, 112, ***<0.0001***	1, 89.1, ***<0.0001***
	Temperature *C:P	1, 8.26, ***0.0064***	1, 4.0, 0.0509	1, 7.77, ***0.0080***

One-Way ANOVAs		Agrobacterium (df, p-values)	Flavobacterium (df, p-value)
		df_{error}=41	df_{error}=41
N:P	Temperature	1, **p<0.05 only at $C:P_R$=1000**	1, **p<0.05 only at $C:P_R$=1000**
	Supply C:P	1, **p<0.05 at all temperatures**	1, **p<0.05 at all temperatures**
C:P	Temperature	1, **p<0.05 only at $C:P_R$=1000**	1, p>0.05 at all $C:P_R$s
	Supply C:P	1, **p<0.05 at all temperatures**	1, **p<0.05 at all temperatures**
