# Supplementary material for: The Effects of Nutrient Imbalances and Temperature on the Biomass Stoichiometry of Freshwater Bacteria
Source: Front Microbiol. 2017 Sep 8;8:1692. doi: 10.3389/fmicb.2017.01692 (PMC5596061; doi:10.3389/fmicb.2017.01692)
Supplement: Supplementary file 4 [file Table4.PDF]

Supplement Table 4. ANOVA results for nucleic acids.

	RNA fg $cell^{-1}$ (df, F, p-value)	DNA fg $cell^{-1}$ (df, F, p-value)
Three-Way ANOVA	df_{error}=93	df_{error}=96
Strain	2, 15.18, ***<0.0001***	2, 21.79, ***<0.0001***
Temperature	1, 6.58, ***0.0120***	1, 0.01, 0.9204
Supply C:P	1, 13.86, ***0.0003***	1, 3.40, 0.0682
Strain*Temperature	2, 0.222, 0.8011	2, 1.41, 0.2501
Strain*C:P	2, 0.227, 0.798	2, 0.82, 0.4418
Temperature *C:P	1, 2.226, 0.139	1, 0.10, 0.7504
Strain*Temperature*C:P	2, 0.43, 0.651	2, 0.06, 0.9433

Two-Way ANOVAs		*Agrobacterium* (df, F, p-value)	*Arthrobacter* (df, F, p-value)	*Flavobacterium* (df, F, p-value)
		df_{error}=30	df_{error}=30	df_{error}=35
RNA fg $cell^{-1}$	Temperature	1, 8.39, ***0.0071***	1, 0.99, 0.328	1, 1.62, 0.2115
	Supply C:P	1, 6.19, ***0.0189***	1, 4.71, **0.038**	1, 4.64, ***0.0383***
	Temperature *C:P	1, 3.19, 0.0846	1, 0.77, 0.388	1, 0.03, 0.8654
DNA fg $cell^{-1}$	Temperature	1, 0.73, 0.4006	1, 1.01, 0.3227	1, 1.04, 0.3155
	Supply C:P	1, 9.69, ***0.0040***	1, 0.49, 0.4908	1, 0.17, 0.6797
	Temperature *C:P	1, 0.02, 0.8948	1, 0.13, 0.7177	1, 0.08, 0.7819
